# Ginsenosides Rh2 and Rg3 exert their anti-cancer effects on non-small cell lung cancer by regulating cell autophagy and choline-phosphatidylcholine metabolism

**DOI:** 10.3389/fphar.2025.1507990

**Published:** 2025-06-12

**Authors:** Zhao Min, Che Er-Xi, Wang Lin-Juan, Wang Lin, Liu Bi-Li, Chen Qiu-Fang

**Affiliations:** ^1^ Department of Pharmacy, The First Affiliated Hospital of Xiamen University, School of Medicine, Xiamen University, Xiamen, China; ^2^ Xiamen Key Laboratory for Clinical Efficacy and Evidence-Based Research of Traditional Chinese Medicine, Xiamen, China; ^3^ Science and Education Division, Women and Children’s Hospital, School of Medicine, Xiamen University, Xiamen, China; ^4^ School of Pharmacy, Yantai University, Yantai, China

**Keywords:** ginsenosides Rh2 and Rg3, autophagy, cellular metabonomics, choline-phosphatidylcholine metabolism, non-small cell lung cancer

## Abstract

**Background:**

Ginseng (Panax ginseng C. A. Meyer) herb itself and its derived preparations (e.g. Shenmai injection) are often prescribed for cancer patients as Traditional Chinese Medicines clinically in China. Ginsenosides Rh2 and Rg3 are two of main active components of ginseng. They have significant cytotoxic effect against non-small cell lung cancer (NSCLC), but the mechanisms are not very clear, especially lack of research on the combination of cell autophagy and metabolism.

**Methods:**

In this study, we investigated the regulatory effects of ginsenosides Rh2 and Rg3 on cellular autophagy and metabolism in non-small cell lung cancer cell lines. Their regulations of cellular autophagy were detected by immunofluorescence, MDC staining, and transmission electron microscopy, while their regulations of cellular metabolism were detected by cellular metabolomics.

**Results:**

Our results showed that ginsenosides Rh2 and Rg3 can significantly induce cell autophagy, and can lead to autophagic cell death through endoplasmic reticulum stress-autophagy axis, similar to ginseng total ginsenosides extract (TGS). They also significantly regulate the cell metabolome at the same time. The regulatory effect of ginsenosides Rh2 and Rg3 on the metabolism of choline-phosphatidylcholine may be the cellular metabolic mechanism of their cytotoxicity. Our findings suggested that ginsenosides Rh2 and Rg3 could induce autophagic cell death and regulate choline-phosphatidylcholine metabolism in NSCLC cells.

**Conclusion:**

This study has a new understanding of the antitumor mechanism of ginsenosides Rh2 and Rg3, and suggests a new direction of studying the pharmacological mechanism of natural active components.

## Graphical Abstract

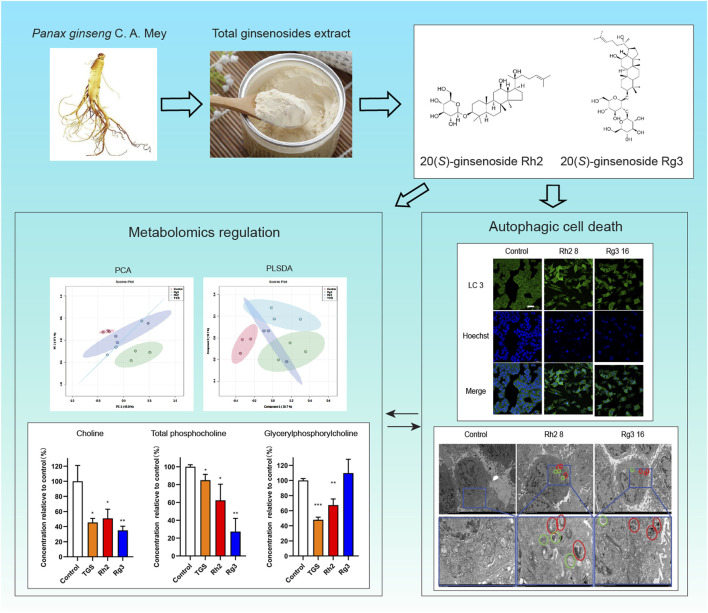

## 1 Introduction

Natural active products are an indispensable part of pharmacy and an important source of modern drug discovery and development. Many natural products and their active components have definite anti-cancer activity; however, their specific mechanisms are mostly unclear, resulting in the limitation of their clinical application (Asma et al., 2022; Newman, 2022). Ginseng (Panax ginseng C. A. Mey.) has been widely used for thousands of years and was often prescribed as a dietary supplement for cancer patients in Asian countries (Liu et al., 2020). Ginseng contains various active components, among which over 100 ginsenosides are the material basis for its various pharmacological activities (Ratan et al., 2021). Various ginsenosides can regulate autophagy in cancer cells through various pathways (Hangui et al., 2023). Ginseng and its preparations (Shenmai injection, for examples) are often prescribed as botanical dietary supplements or medicinal product for cancer patients in China, which can regulate the immune function of patients and has a certain auxiliary anticancer effect (Kang and Min, 2012; Tao et al., 2023; Zhao et al., 2019). However, their effects on autophagy in cancer cells and cell metabolism are still unclear.

Our previous studies have found that total ginsenosides extract (TGS) induce autophagic cell death in NSCLC cells through activation of endoplasmic reticulum stress (ER stress) (Zhao et al., 2019). Ginsenosides Rh2 and Rg3 have similar regulations on autophagy to TGS, and they have significant cytotoxicity to NSCLC. The regulation of ginsenosides Rh2 and Rg3 on autophagy in different tumors are different (Bian et al., 2019; Zheng et al., 2017). Whether ginsenosides Rh2 and Rg3 can play an anti-NSCLC role by regulating cell autophagy and cell metabolism is still unknown. In the present research, we studied the cytotoxicity of ginsenosides Rh2 and Rg3 on NSCLC cell and found that ginsenosides Rh2 and Rg3 could upregulate cell autophagy flux, further leading to autophagic cell death. Further investigations demonstrated that ginsenosides Rh2 and Rg3 have significant regulatory effect on cell metabolism, their regulation of choline-phosphatidylcholine metabolism in cells may be closely related to autophagy induction. Our study discloses the characteristics of ginsenoside Rh2 and Rg3 induced cell death and their regulations on choline-phosphatidylcholine metabolism.

## 2 Materials and methods

### 2.1 Reagents and cell culture

Ginsenosides monomers (purity 98%, 20(*S*)-ginsenoside Rh2, CAS number: 7821-33-2; 20(*S*)-ginsenoside Rg3, CAS number:14,197-60-5) and total ginsenosides extract (TGS) were bought from Jilin University (Changchun, China). The composition of TGS has been tested experimentally (Hao et al., 2010). Ginsenosides Rh2 and Rg3 were dissolved in DMSO at a series of concentrations. TGS was dissolved in RPMI 1640 medium and filtered through a 0.22 μm membrane, with a final concentration of 100 mg/mL. 3-methyladenine (3-MA,Cat.No.M9281), Chloroquine (CQ,Cat.No.C6628), DMSO (Cat.No.D4540), monodansylcadaverine (MDC,Cat.No.30432), and rapamycin (Rapa,Cat.No. 553210) were purchased from Sigma Aldrich (St Louis, MO); Lipofectamine 3,000 (Cat.No.L300015), ATG7 siRNA (Cat.No.HSS116182), BECN siRNA (Cat.No. HSS112741) and control siRNA (medium GC,Cat.No.12935300) were purchased from Invitrogen Trading Shanghai Co. (Shanghai, China). Cell Counting Kit 8 (CCK-8, Cat.No. K009-500) was purchased from Zeta life (California, United States of America). RPMI 1640 medium (Cat.No.C11875500BT) was from GIBCO (California, United States of America). Fetal bovine serum (FBS,Cat.No.FCS 500) was purchased from ExCell Biotechnology Co., Ltd (Shanghai, China). BCA Protein Assay Kit (Cat.No.0012), Hoechst 33,342 (Cat.No.C1022), Phenylmethanesulfonyl fluoride (PMSF, Cat. No. ST507-10 mL), Phosphate buffered saline (PBS, Cat. No. ST448-1L), RIPA lysis buffer (Cat.No. P0013B),Triton X-100 (Cat.No.ST795) and other reagents were from Beyotime Biotechnology (Shanghai, China).

Human NSCLC A549 and PC-9 cell lines were bought from the American Type Culture Collection (ATCC) (Maryland, United States of America) and cultured in RPMI 1640 medium containing 10% FBS, 100 μg/mL streptomycin and 100 U/mL penicillin at 37°C with 5% CO_2_.

### 2.2 Cell proliferation assay

96 well plate was used to culture A549 and PC-9 cells, and when the density reaches 80%, cells were treated with ginsenosides Rh2, Rg3 and TGS with indicated concentrations for 24 or 48 h, cytotoxicity was tested using CCK-8 kit according to instruction. The absorbance at 450 nm was measured using a BioTek, Synegy H1 Hybrid Reader (Vermont, United States of America), cell inhibition rates are displayed as percentages compared to the control group.

### 2.3 Cell morphological observation

NSCLC cells with 80% confluence in 12-well plates were treated with ginsenoside Rh2, Rg3 and TGS for 12 h, cells were washed with PBS and observed using Leica DMI3000B fluorescence microscope (Bensheim, Germany) in the brightfield.

### 2.4 Western blotting analysis

A549 cells were treated with ginsenoside Rh2, Rg3 and TGS, and the total protein levels in cell lysates were measured using protein quantification kit. Boiled cell lysates contained 60 μg total protein were loaded to 8%–12% gradient polyacrylamide gels (Bis-Tris Midi Gel, Life Technologies, United States of America) and analyzed by Western blotting with corresponding antibodies. The same blots were tested with GAPDH antibody to normalize protein load. Developing photos of protein blottings are adjusted and cut using Image Lab software.

The primary antibodies anti-ATF4 (Cat.No.11815), anti-ATG5 (Cat.No.12994T), anti-ATG7 (Cat.No.8558), anti-BECN (Cat.No.4122S), anti-Bip (Cat.No.3177), anti-CHOP (Cat.No.5554),anti-LC3B (Cat.No.2775), anti-PERK (5683T) antibodies and secondary antibodies (Cat.No.7076/7074) were purchased from Cell Signaling Technology (Danvers, MA, United States of America), anti-IRE1 antibody (Cat.No.ab124945) was purchased from Santa Cruz Biotechnology (Dallas, MA,United States of America) and anti-GAPDH antibody (Cat.No.AP0063), anti-p62 (Cat.No.AF5312) was purchased from Beyotime Biotechnology (Shanghai, China).

### 2.5 Immunofluorescence

0.17 mm glass-bottom dishes were used to culture A549 cells, and when the density reaches 80%, cells were treated with agents for the indicated times, then cells were fixed with 4% formaldehyde for 30 min at 4°C, permeabilized with PBS/T (PBS containing 0.1% Tween-20) for 20 min at room temperature, blocked with 5% w/v BSA in PBS/T for 1 h at 37°C and then incubated with LC3B antibody overnight at 4°C, after washed with PBS/T for 4 times, cells were incubated with fluorescent secondary antibody for 1 h and Hoechst 33,342 for 0.5 h in the dark, then cells were washes and observed with a Leica TCS SP8 confocal fluorescence microscope (Bensheim, Germany).

### 2.6 MDC (Monodansylcadaverine) Staining

12-well plates were used to culture A549 cells, and when the density reaches 80%, cells were treated with ginsenosides Rh2 and Rg3 and other agents for 12 h, then cells were cultured with 50 μM MDC in PBS for 30 min at 37°C, after washed with PBS for 4 times, cells were observed with a Leica DMI3000B fluorescence microscope (Bensheim, Germany).

### 2.7 Transmission electron microscope observation

A549 cells were treated with indicate agents for 12 h, then cells were fixed with 2.5% glutaraldehyde and 1% osmium tetroxide successively, and dehydrated with a range of concentrations of acetone, after penetrated and embedded, cells were sliced on ultramicrotome and electronic dyed with sodium acetate and lead citrate, finally, slices were observed with Hitachi HT7800 transmission electron microscope (Tokyo, Japan).

### 2.8 Gene knockdown

All transfections were performed using Lipofectamine 3,000 according to its instructions. Cells were transfected with ATG7 siRNA (15 and 30 nM), BECN siRNA (25 and 50 nM), or negative control (medium GC) at concentrations of 20 nM siRNA. The sequences of siRNA duplex for ATG7 siRNA and BECN siRNA are shown as Table 1.

**TABLE 1:** The sequences of siRNA duplex for ATG7 siRNA and BECN siRNA.

Gene	Sense strand	Antisense strand
ATG7	5’-GGUUUGGACGAAUUCCAACUUGUUU-3’	5’-AAACAAGUUGGAAUUCGUCCAAACC-3’
BECN	5’-CCACUCUGUGAGGAAUGCACAGAUA-3’	5’-UAUCUGUGCAUUCCUCACAGAGUGG-3’

### 2.9 Quantitative real time PCR assay

Total RNA in cells was extracted using RNAiso Plus reagent (TaKaRa Biotechnology Co., Ltd, Dalian, China) according to the manufacturer’s instructions. The concentrations of RNA were tested by measuring their absorbance at 260 and 320 nm. Complementary DNA was reverse transcribed from 500 ng total RNA using FastKing RT Kit (Tiangen Biochemical Technology (Beijing) Co., Ltd). Quantitative real time PCR analysis was performed using Super Real PreMix Plus (SYBR green) (Tiangen Biochemical Technology (Beijing) Co., Ltd) in a reaction volume of 15 μL on LightCycler 480 II RT-PCR system (Basel, Switzerland). The annealing was carried out at 60°C for 30 s. Relative gene expression analysis was calculated using the 2 (^−ΔΔCt^) method with GAPDH as a control. Each experiment was carried out in duplicates. The primer sequences are shown in Table 2. (Forward 5’-3’, Reverse 5’-3’).

**TABLE 2:** Primer sequences for quantitative RT-PCR Assay.

Gene	Forward primer	Reverse primer
GAPDH	CCAGGGCTGCTTTTAACTC	GCTCCCCCCTGCAAATGA
ATF4	GAAGGTCATCTGGCATGGTT	AGTCCCTCCAACAACAGCAA
CHOP	ACCAAGGGAGAACCAGGAAACG	TCACCATTCGGTCAATCAGAGC
Bip	GCTATTGCTTATGGCCTGGA	CGCTGGTCAAAGTCTTCTCC
BECN	GTC​GCT​GAA​GAC​AGA​GCG​AT	CGA​TGC​TCT​TCA​CCT​CGG​G
ATG 5	TGG​ATG​GGA​TTG​CAA​AAT​GAC​A	AGT​AAG​ACC​AGC​CCA​GTT​GC
ATG 7	AGT​GCC​TTG​GAT​GTT​GGG​TT	AGA​CAG​AGG​GCA​GGA​TAG​CA

### 2.10 Metabolomic analysis

#### 2.10.1 Sample preparation

A549 cells were cultured in 6-well plates and treated with TGS, ginsenosides Rh2 and Rg3 for 12 h. Cells were collected with 200 μL methanol and 3 times freeze-thaw cycles in a refrigerator at – 80°C. After which samples were centrifuged at 15,000 rpm for 10 min and take the supernatant. Take 10 μL from each sample to make quality control samples by mixing. Four times the volume of ice methanol extract (4°C) containing Internal Standard (berberine, 200 ng/mL) was added to precipitate protein in the sample, vortex for 1 min, 15,000 rpm, and centrifuge at 4° C for 10 min. Take the supernatant and add 60 μL 90% (vol/vol) (methanol: water) to redissolved, vortex for 1 min, 15,000 rpm, centrifugation at 4 °C for 10 min, take the supernatant for injection.

#### 2.10.2 Liquid chromatography-mass spectrometry analysis method

The chromatographic column used is Accucore HILIC column (100 × 2.1 mm 2.6 μm). The aqueous phase (A) is an aqueous solution containing 5 mM ammonium formate, and the organic phase (B) is acetonitrile: methanol: water (90:5:5, v/v). The flow rate is 0.40 mL/min, and the column temperature is 40° C. The gradient elution method is used for chromatographic separation which was shown in Table 3, and the injection volume is 5 μL. Q Active Orbitrap uses ESI ion source, positive ion mode, full MS-dMS2 mode to collect data, and the m/z scanning range is set to 50-750 Da. The injection voltage of the ion source is +3.5 kV and −2.5 kV respectively, the capillary temperature is 350°C, the heater temperature is 300°C, the sheath gas flow rate is 35 arb, and the auxiliary gas flow rate is 10 arb. The resolutions of Full MS and MS2 are 35,000 and 17,500 (FWHM) respectively, and the NCE is set to 15 V, 30 V and 45 V.

**TABLE 3:** Gradient elution procedure.

Time (min)	Mobile phase	
	A (%)	B (%)
0	15	85
1	15	85
4	35	65
4.5	35	65
4.51	15	85
6	15	85

The original data was processed by Compound Discover 3.1 (Thermofisher, United States of America), and the mass deviation was set to 5 ppm. The compounds were initially identified, the obtained chromatographic peaks were automatically integrated and manually checked, and the retention time, peak area ratio, substance name, molecular formula and molecular weight matched by the software were exported. Export the data to metabianalyst for multivariate statistical analysis, and analyze the pathway of different metabolites.

### 2.11 Statistical analysis

Data were showed as mean ± standard deviation (SD). Prism 9.3 statistical software was used for the analysis. The two-tailed Student t-test was used to reveal statistical significance. *P* < 0.05 was considered statistically significant.

## 3 Results

### 3.1 Ginsenosides Rh2 and Rg3 have significant cytotoxicity on NSCLC

**FIGURE 1:**
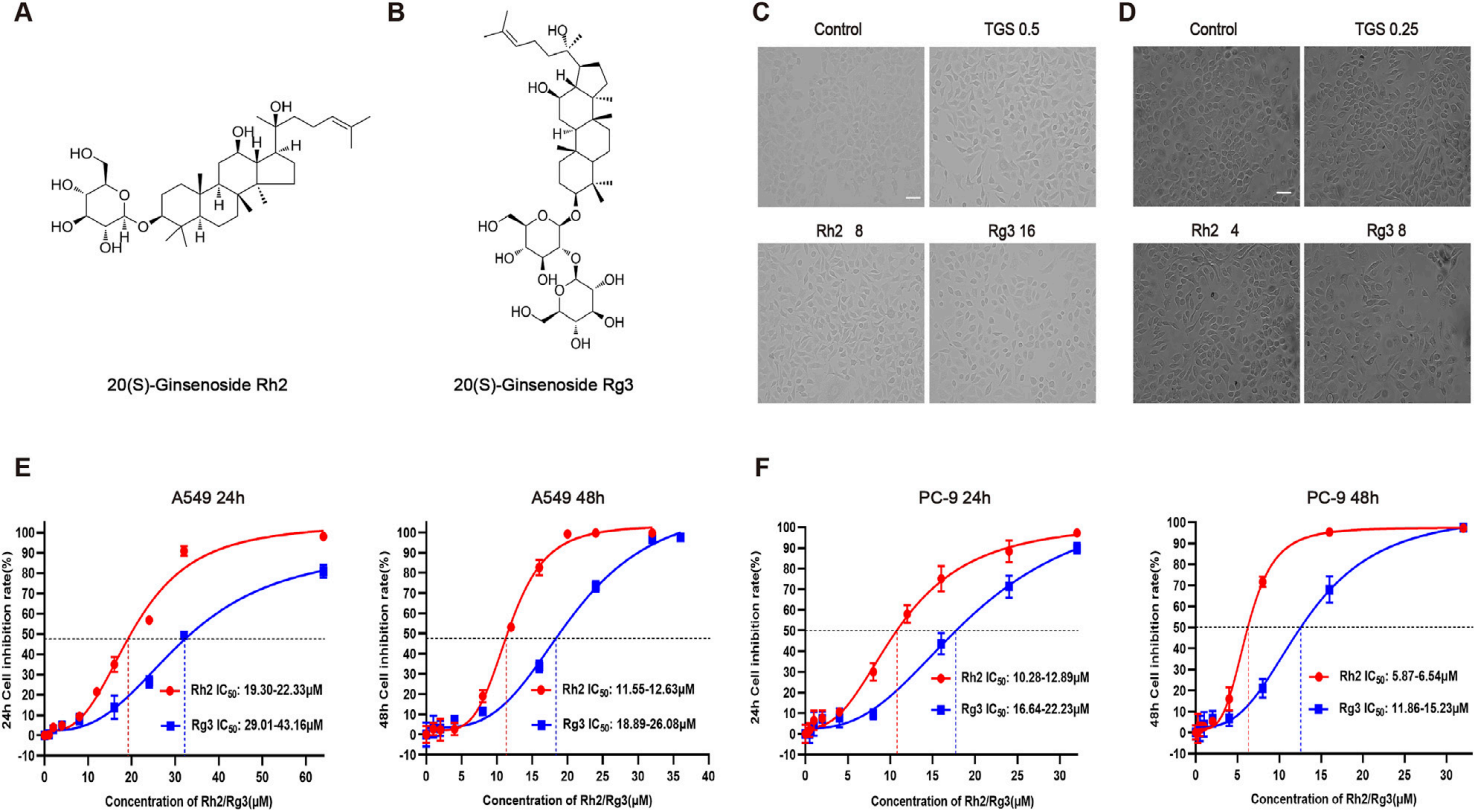
Ginsenosides Rh2 and Rg3 have significant cytotoxicity on NSCLC. **(A,B)** Chemical structure of 20(S)-ginsenoside Rh2 and 20(S)-ginsenoside Rg3. **(C) (D)** Cell morphological observation after treated with 0.5 or 0.25 μg/mL TGS (TGS 0.5, TGS 0.25), 4 or 8 μM Rh2 (Rh2 4, Rh2 8) and 8 or 16 μM Rg3 (Rg3 8, Rg316) for 24 h, A549 cells **(C)**, PC-9 cells **(D)**. Scale bars, 20 μm. **(E,F)** Survival curves describing the viability of A549 cells **(E)** or PC-9 cells **(F)** treated with a series of concentrations of ginsenosides Rh2 and Rg3 for 24 or 48 h. The data was expressed as cell inhibition relative to untreated controls and represented the average value ±S.E.M. n = 3 for each group.

Our previous studies have found that TGS induce autophagic cell death in NSCLC cells through activation of ER stress. In order to further reveal the active monomer components of autophagic regulation of TGS, we screened a variety of ginsenoside monomers, and the results showed that ginsenosides Rh2 and Rg3 have significant cytotoxicity to NSCLC among numerous ginsenoside monomers (Figures 1E,F). Cell morphological observation showed that A549 and PC-9 cells changed from cobblestone shape to mesenchymal-like and elongated spindle shape after treated with ginsenosides Rh2 and Rg3 (Figures 1C,D), cell adhesion was reduced and exhibited an autophagic cell death-like morphology. These results indicate that the active monomer components of autophagic death of TGS may be ginsenosides Rh2 and Rg3. Ginsenosides Rh2 and Rg3 also exhibit similar cytotoxicity in other cell lines, as shown in Supplementary Figure S1.

### 3.2 Ginsenosides Rh2 and Rg3 increase autophagy flux in NSCLC A549 cells

**FIGURE 2:**
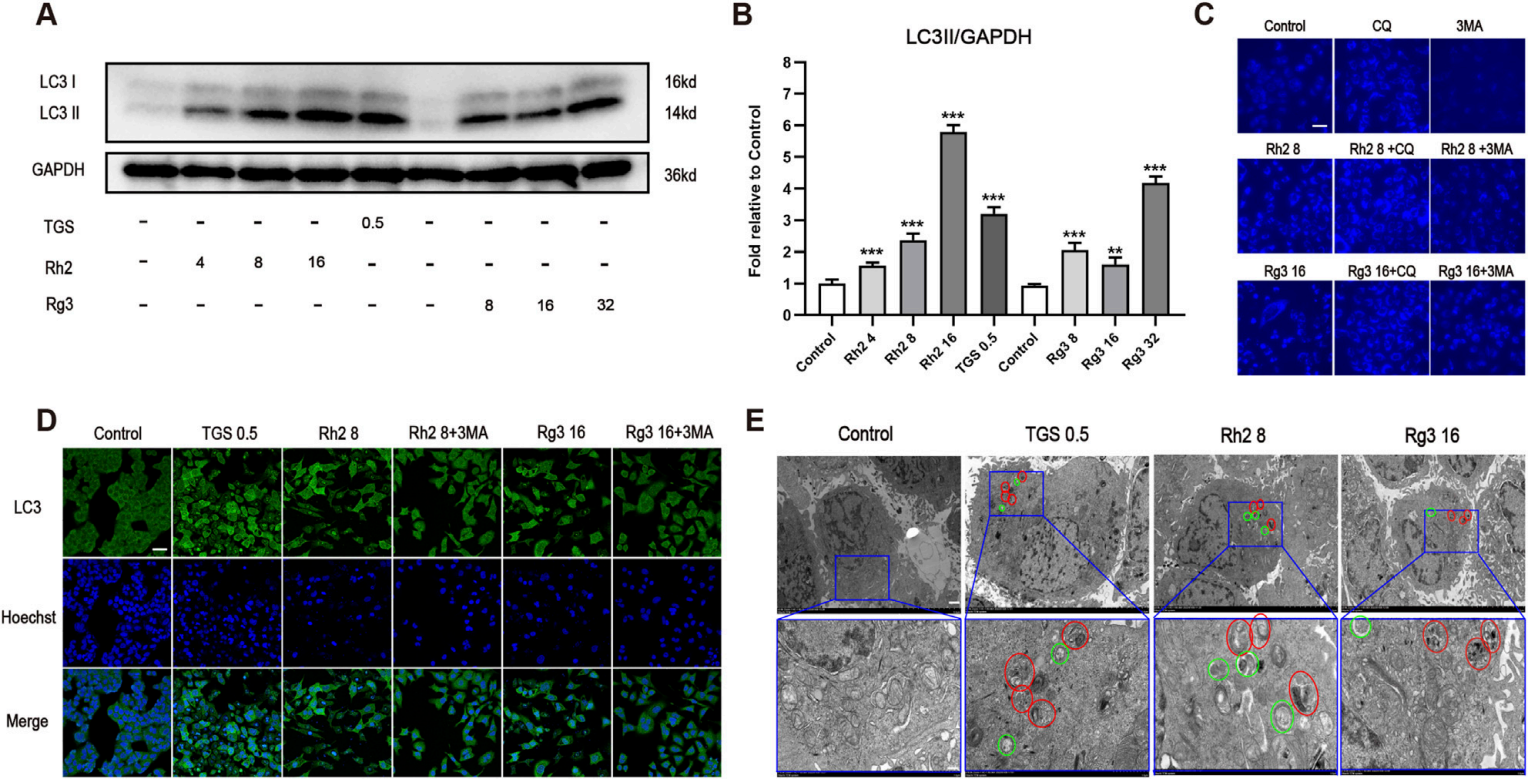
Ginsenosides Rh2 and Rg3 increase autophagy flux in NSCLC A549 cells. **(A)** Immunoblot analysis with the LC3B antibody on cell lysates treated with TGS, ginsenosides Rh2 and Rg3 for 12 h, GAPDH serves as a loading control. **(B)** The semi-quantitative results of LC3II/GAPDH of **(A)** using the Volume Tool in Image Lab software. **P < 0.01, ***P < 0.001 *versus* control. **(C)** A549 cells were treated with TGS, ginsenosides Rh2 and Rg3, with or without 5 μM chloroquine (CQ), 5 mM 3-methyladenine (3-MA) for 12 h, autophagosome formation was indicated by the blue stains of MDC detected under 488 nm. Experiment was performed in triplicate and representative images were showed. Scale bars, 20 μm. **(D)** Immunofluorescence analysis of LC3 puncta formation in cells treated with TGS, ginsenosides Rh2 and Rg3 for 12 h, Hoechst 33,342 was used to stain the nuclei. Experiment was performed in triplicate and representative images were showed. Scale bars, 20 μm. **(E)** The transmission electron microscopy images of the A549 cells treated with TGS, ginsenosides Rh2 and Rg3 for 12 h, representative images were showed. Circles in green represented autophagosomes, and red ones indicated autolysosomes. Scale bars, 10 μm, insets 3 μm.

To confirm the type of cell death induced by ginsenosides Rh2 and Rg3, we examined whether ginsenosides Rh2 and Rg3 induce cell apoptosis. The results showed that ginsenosides Rh2 and Rg3 can induce a small amount of cell apoptosis, and the expression and activation levels of apoptotic proteins are also very limited. The results are shown in Supplementary Figure S3. To confirm the regulatory effects of ginsenosides Rh2 and Rg3 on cell autophagy, we tested their regulations on the level of autophagic marker proteins and the production of intracellular autophagosomes and autophagic lysosomes. The results showed that ginsenosides Rh2 and Rg3 could significantly upregulate the autophagy flux (Figures 2A,B,D). The detection results of dual fluorescence mRFP-GFP-LC3 are similar to those of immunofluorescence, as shown in Supplementary Figure S2. Through MDC staining and electron microscopy, we found significant appearance and accumulation of autophagosomes and autophagic lysosomes in cells (Figures 2C,E). These results confirm that ginsenosides Rh2 and Rg3 could significantly increase autophagy flux in NSCLC A549 cells.

### 3.3 Ginsenosides Rh2 and Rg3 induce autophagic cell death in NSCLC A549 cells through activation of endoplasmic reticulum stress

**FIGURE 3:**
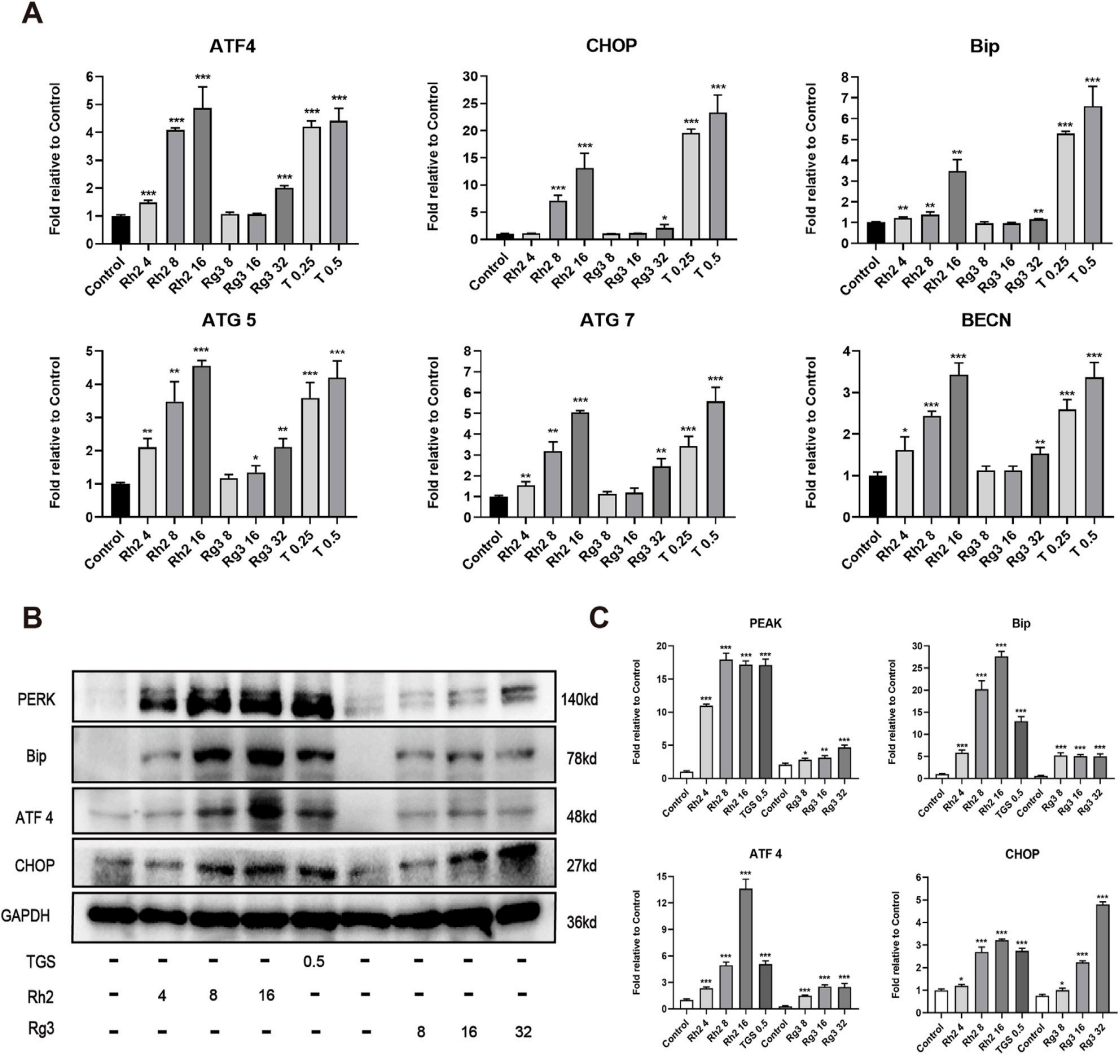
Ginsenosides Rh2 and Rg3 induces cell death in NSCLC A549 cells through activation of endoplasmic reticulum stress. **(A)** Quantitative RT-PCR analysis of mRNA levels of ATF4, CHOP, Bip, ATG5, ATG7, BECN in A549 cells treated with TGS, ginsenosides Rh2 and Rg3 for 12 h, mRNA levels were relative to control, GAPDH serves as a housekeeping control. *P < 0.05, **P < 0.01, ***P < 0.001 *versus* control. **(B)** Immunoblot analysis with the indicated antibodies on cell lysates treated with TGS, ginsenosides Rh2 and Rg3 for 12 h, GAPDH serves as a loading control. **(C)** The semi-quantitative results of target proteins/GAPDH of **(B)** using the Volume Tool in Image Lab software. *P < 0.05, **P < 0.01, ***P < 0.001 *versus* control.

**FIGURE 4:**
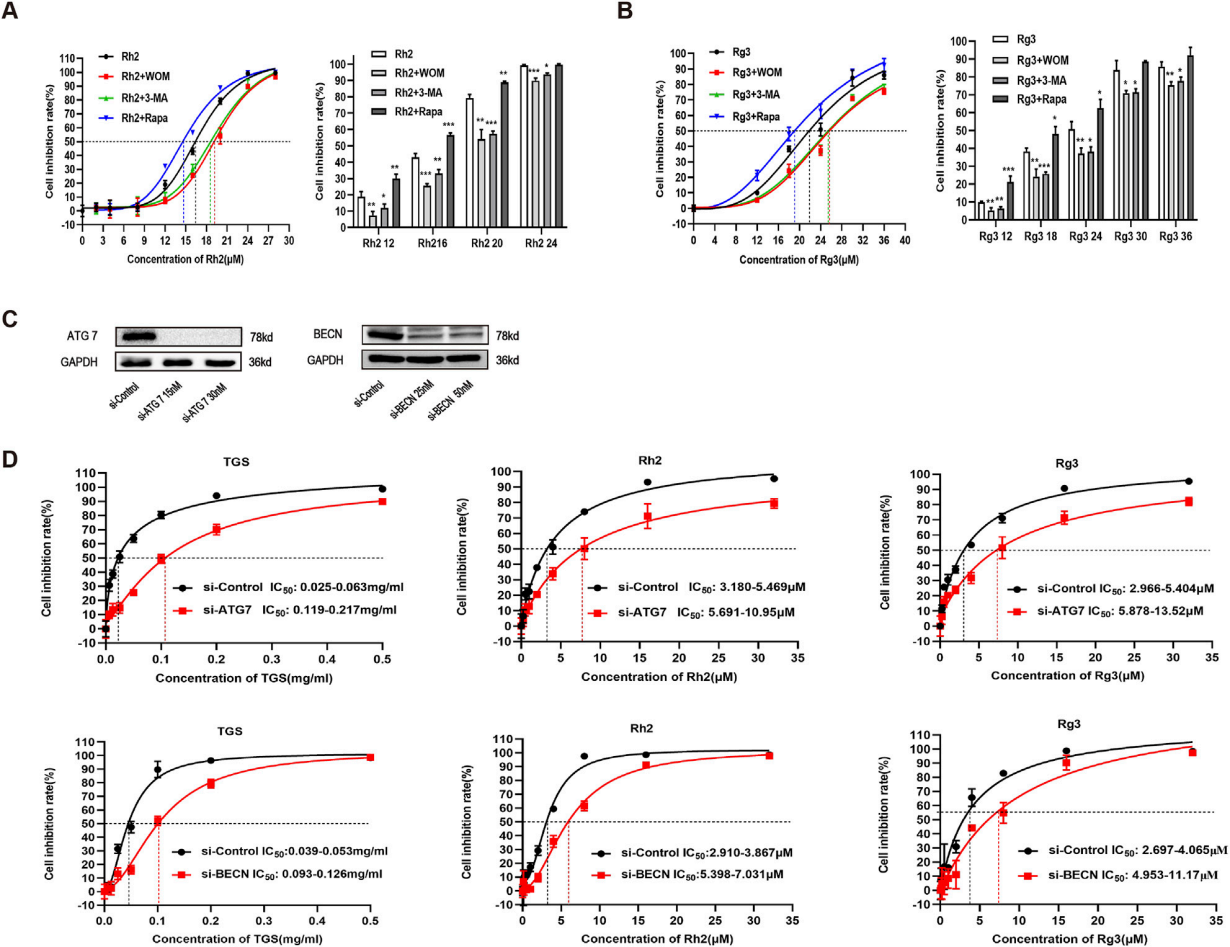
Ginsenosides Rh2 and Rg3 induced cell death in NSCLC A549 cells are autophagy dependent. **(A,B)** A549 cells were treated with ginsenosides Rh2 **(A)** and Rg3 **(B)** with or without 1 μM wortmannin (WOM), 5 mM 3-methyladenine (3-MA) for 48 h, cell viability was detected with CCK-8, *P < 0.05, **P < 0.01, ***P < 0.001 *versus* ginsenosides Rh2 and Rg3 alone. **(C)** verification of ATG7 and BECN knockdowns in A549 cells. **(D)** Effects of TGS, ginsenosides Rh2 and Rg3 treatment (48 h) on cell viability transfected with control siRNAs (si-Control) or ATG 7- and BECN selective siRNAs (si-ATG7 and si-BECN), *P < 0.05, **P < 0.01 *versus* si-control.

To explore whether the regulation of ginsenosides Rh2 and Rg3 on autophagy is related to ER stress - autophagy axis, we detected the mRNA and protein levels of various ER stress - autophagy related genes. The results showed that the mRNA and protein levels of various ER stress - autophagy related proteins were upregulated (Figures 3A–C). Autophagy inhibitors and silence of important proteins of autophagy pathway ATG7 and BECN can significantly reverse the cytotoxicity of ginsenosides Rh2 and Rg3 on NSCLC A549 cells (Figures 4A–D. These results indicate that the cytotoxicity of ginsenosides Rh2 and Rg3 are stimulated by ER stress, and are autophagy dependent.

### 3.4 Ginsenosides Rh2 and Rg3 have significant regulatory effects on cell metabolomics and choline -phosphatidylcholine metabolism

**FIGURE 5:**
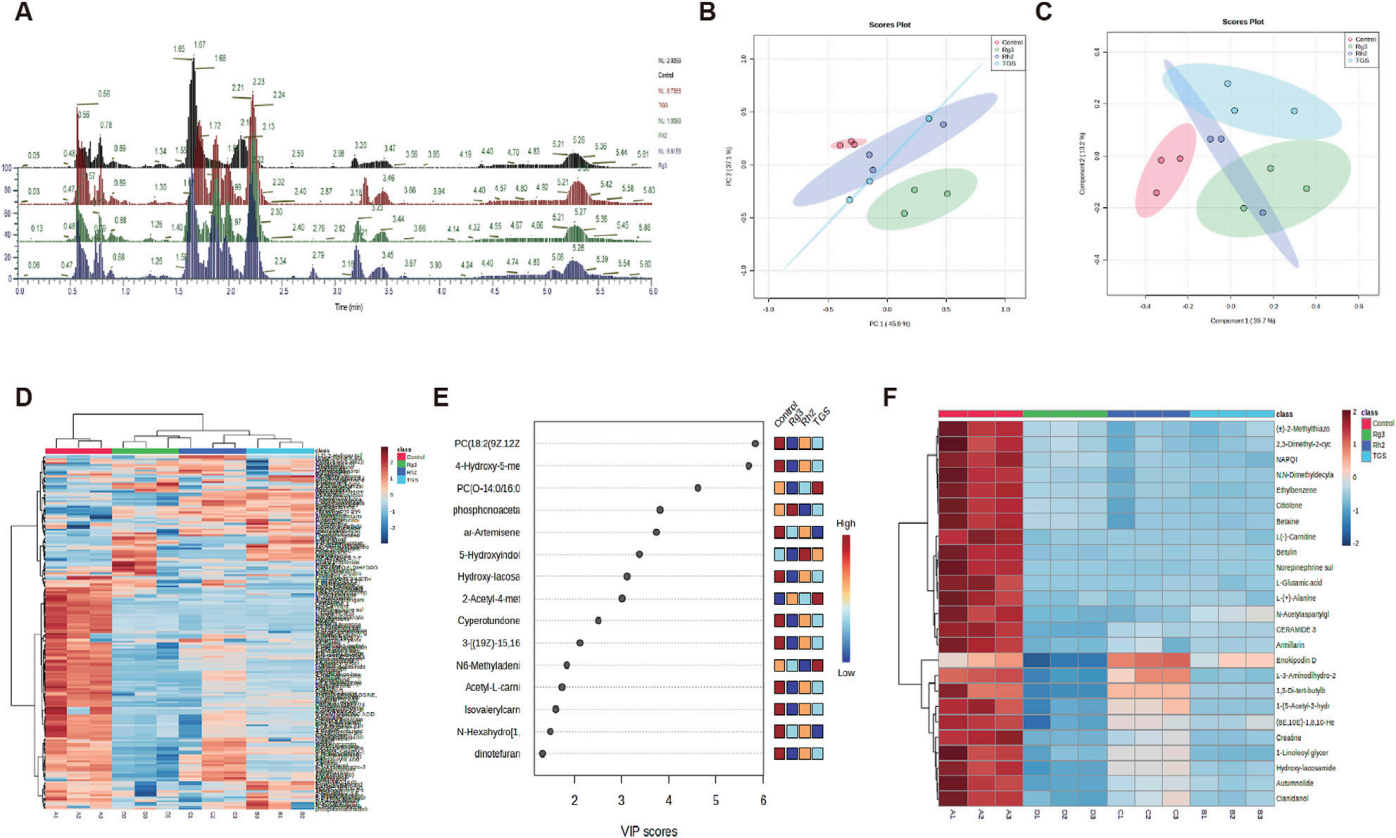
Metabolomic analysis of ginsenosides Rh2 and Rg3 on A549 cells. **(A)** Total ion current spectrum information of TGS (0.5 μg/mL), ginsenosides Rh2 (8 μM) and Rg3 (16 μM) treatments. **(B**–**F)**. PCA, PLSDA, total heatmap, important compounds and refined heat map of TGS, ginsenosides Rh2 and Rg3 treatments.

**FIGURE 6:**
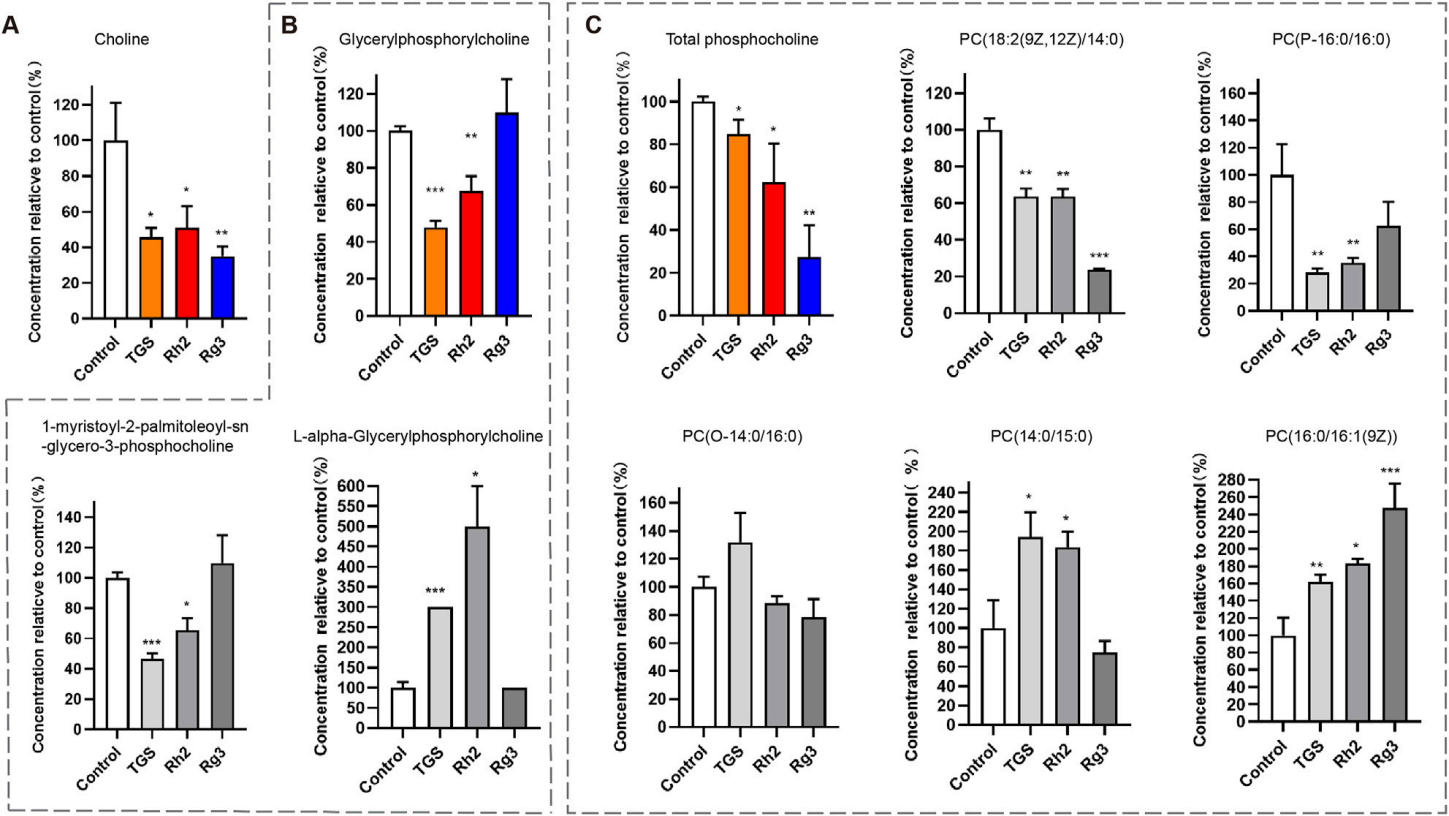
Regulation of ginsenosides Rh2 and Rg3 on Choline Metabolism in Cells A549 Cell was treated with 0.5 μg/mL TGS, 8 μM Rh2 and 16 μM Rg3, choline and various phosphorylcholines were detected with LC-MS/MS. *P < 0.05, **P < 0.01, ***P < 0.001 *versus* control. **(A)** Choline level, **(B)** Total glycerylphosphorylcholine level (color panel), two kinds of glycerylphosphorylcholines (2 gray panels). **(C)** Total phosphatidylcholine level (color panel), five kinds of phosphatidylcholine (PC, 5 gray panels).

Due to the close relationship between autophagy and cell metabolism, we then investigated the regulatory effect of ginsenosides Rh2 and Rg3 on cell metabolism. Analysis of metabonomics results shows that the regulatory effect of TGS on cell metabolism is roughly similar to the comprehensive effect of ginsenosides Rh2 and Rg3. Similar metabolic pathway regulation focuses on choline-phosphatidylcholine metabolism. Similar to TGS, ginsenosides Rh2 and Rg3 significantly regulate the metabolism of choline-phosphatidylcholine in cells and reduce the intracellular choline and phospha-tidylcholine, indicating that the cytotoxicity of ginsenosides Rh2 and Rg3 on NSCLC cells may be related to their regulations of choline metabolism (Figures 5, 6). We simultaneously detected the levels of choline and phosphatidylcholine in non-small cell lung cancer cells after ATG seven was silenced. The results showed that after autophagy was inhibited, the levels of choline and phosphatidylcholine in cells increased, as shown in Supplementary Figure S4. This result is consistent with the induction of autophagy by total ginsenosides extract, ginsenosides Rh2 and Rg3, leading to a decrease in choline and phosphatidylcholine levels. The association between autophagy and choline phosphatidylcholine metabolism still needs further investigation.

## 4 Discussion

According to the latest statistical data, lung cancer is still the cancer with the largest number of new cases in the world. Meanwhile, as the disease with the highest mortality rate among cancers, lung cancer causes more than 350 deaths every day—more than the sum of breast cancer, prostate cancer and pancreatic cancer, and 2.5 times that of colorectal cancer (the second leading cause of cancer death in the United States) (Siegel et al., 2022). Non-small cell lung cancer (NSCLC) accounts for about 85% of lung cancer and is the main type of lung cancer (Ganti et al., 2021). In recent years, the treatment of NSCLC has made great progresses, and the emergence of a variety of new medicines have brought hope for the treatment of NSCLC. The combination of natural active products and chemotherapy drugs is a clinical method to improve curative effect and overcome drug resistance, and the efficacy has been verified in clinical practice (Hu et al., 2020; Zang et al., 2020).

ER stress is a kind of cellular response to protein misfolding, which has profound effect on cell survival and death. A large number of studies have shown that ER stress is involved in many diseases, such as central nervous system diseases, inflammation, cancer, and so on (Marciniak et al., 2022). In cancer cells, ER stress may activate autophagy, degrade misfolded proteins in cells and reuse them, which is a way to survive under stress, therefore, ER stress has become a therapeutic target for the treatment of many cancers (King and Wilson, 2020; Xu et al., 2022). Autophagy is an evolutionarily conserved membrane transport and degradation process, which operates at basal levels under normal conditions as a means of degrading cytosolic proteins and organelles. In the absence of nutrients or growth factors, autophagy acts as a self-limited survival mechanism by recycling cytoplasmic substance. However, the phenomenon of autophagy is frequently observed in cells undergoing programmed cell death suggesting that autophagy may be involved. The cell death caused by autophagy is called autophagic death, which was also called Type II cell death. Therefore, autophagy regulates cell fate differently under different circumstances (Gao et al., 2022). ER stress is a potent trigger for autophagy, the relationship between ER stress and autophagy is intertwined and has been reviewed (Mizushima and Levine, 2020; Yu, 2020).

Our previous studies have found that TGS induce autophagic cell death in NSCLC cells through activation of ER stress-autophagy axis, however, the active monomer components are still unknown (Zhao et al., 2019). In this study, we first screened the cytotoxicity and autophagy regulation of various ginsenoside active monomers in NSCLC, and found that ginsenosides Rh2 and Rg3 induce cell death in concentration and time dependent manners in NSCLC cells, meanwhile, treated cells with ginsenosides Rh2 and Rg3 will cause cells to display a morphology of autophagic cell death. Therefore, we further focused on the efficacy and mechanism of ginsenosides Rh2 and Rg3 on autophagic cell death in NSCLC. Ginsenosides Rh2 and Rg3 could significantly induce the production and accumulation of autophagic membrane protein LC3II, indicating that they can significantly upregulate autophagy flux. Through MDC staining, immunofluorescence and electron microscopy, we verified the induction of cell autophagy by ginsenosides Rh2 and Rg3. Many studies have shown that ginsenosides Rh2 and Rg3 could inhibit cell proliferation, differentiation, invasion, and induce cell cycle inhibition and apoptosis, however, the regulatory effect of ginsenosides Rh2 and Rg3 on autophagy are rarely studied (Sun et al., 2017; Yi et al., 2020). Excessive autophagy could lead to autophagic cell death, which is also the mechanism and target of anticancer drugs (Patra et al., 2022; Liu and Levin, 2015). Therefore, we further studied the effect of ginsenosides Rh2 and Rg3 on ER stress-autophagy axis in NSCLC cells. The results showed that ginsenosides Rh2 and Rg3, similar to TGS, could significantly cause the upregulation of various proteins related to ER stress and autophagy. The inhibition of autophagy and the silencing of autophagy related genes could significantly reverse the cytotoxicity of ginsenosides Rh2 and Rg3 in NSCLC cells, indicating that cell death caused by ginsenosides Rh2 and Rg3 are autophagic dependent, which are autophagic cell death. Ginsenosides Rh2 and Rg3 have significant regulatory effects on cell metabolism, and their regulation of cell material and energy metabolism may be the mechanism of their treatment of diseases (Lee et al., 2020; Liu et al., 2020a). In order to further verify that the autophagic cell death effect of TGS mainly comes from ginsenosides Rh2 and Rg3, we analyzed the regulatory effect of TGS and ginsenosides Rh2 and Rg3 on cell metabonomics, the results showed that the regulation of ginsenosides Rh2 and Rg3 on cell metabonomics could roughly simulate that of TGS, indicating that ginsenosides Rh2 and Rg3 maybe the main active components of TGS to induce autophagic cell death. Similar metabolic pathway regulations focus on choline-phosphatidylcholine metabolism, indicating that the regulation of ginsenosides Rh2 and Rg3 on autophagy may be related to their regulation of choline metabolism. Choline and phosphatidylcholine can transform each other in cells and phosphatidylcholine is the major cellular membrane component. Therefore, the metabolism of choline-phosphatidylcholine in cancer cells is abnormally active. Their direct uptake and *De novo* synthesis are more and faster than those in normal cells to meet the rapid proliferation of cancer cells (RF et al., 2022). The regulatory effect of ginsenosides Rh2 and Rg3 on the metabolism of choline-phosphatidylcholine may be the cellular metabolic mechanism of their cytotoxicity. At present, the interaction between choline metabolism and autophagy is still very rare. Study has shown that choline can inhibit cardiomyocyte autophagy induced by myocardial ischemia-reperfusion in rats by activating the Akt/mTOR signaling pathway (Hang et al., 2018). This indicates that choline has a negative regulatory effect on autophagy of cardiomyocytes. In breast cancer MCF-7 cell lines, downregulation of choline kinase will lead to significant downregulation of phosphatidylcholine level, which can induce autophagy of cells (Kim et al., 2015). These results consistent with our findings that ginsenosides Rh2 and Rg3 reduce choline levels in NSCLC and induces autophagic death in this article.

## 5 Conclusion

In this paper, our findings suggested that ginsenosides Rh2 and Rg3 could induce autophagic cell death and regulate cell metabolomics in NSCLC cells, the regulation of ginsenosides Rh2 and Rg3 on autophagy may be related to their regulation of choline metabolism. This study has a new understanding of the antitumor mechanism of ginsenosides Rh2 and Rg3, and suggests a new direction of studying the pharmacological mechanism of natural active components. The summary of this article is shown in Graphical Abstract.

## Data Availability

The original contributions presented in the study are included in the article/Supplementary Material; further inquiries can be directed to the corresponding authors.

## Supplementary material

SUPPLEMENTARY FIGURE S1
Ginsenosides Rh2 and Rg3 have significant cytotoxicity on human breast ductal carcinoma cell line BT-474 and human colon adenocarcinoma cell line SW480. Survival curves describing the viability of human breast ductal carcinoma BT-474 cells **(A)** or human colon adenocarcinoma SW480 cells **(B)** treated with a series of concentrations of ginsenosides Rh2 and Rg3 for 48 h. The data was expressed as cell inhibition relative to untreated controls and represented the average value ±S.E.M. n = 6 for each group.

SUPPLEMENTARY FIGURE S2
Ginsenosides Rh2 and Rg3 increase autophagy flux in NSCLC A549 cells. **(A)** Dual-fluorescence mRFP-GFP-LC3 probe was used to determine the effect of ginsenoside Rh2 and Rg3 on autophagic flux. **(B)** Relevant statistical data of the LC3 puncta numbers in panel **(A)**.

SUPPLEMENTARY FIGURE S3
Ginsenosides Rh2 and Rg3 induce minimal cell apoptosis. **(A)** Apoptosis assay kit was used to detect the apoptosis of ginsenoside Rh2 and Rg3 in A549 cells; **(B)** Statistics of apoptosis data in **(A)**. ***P* < 0.01, ****P* < 0.001 *versus* control. **(C)** Immunoblot detection of apoptotic protein expression of A549 cells after treated with ginsenoside Rh2 and Rg3 for 24 h.

SUPPLEMENTARY FIGURE S4
Detection of choline and phosphatidylcholine levels after ATG 7 silencing. **(A)** Verification of ATG7 knockdowns in A549 cells. **(B,C)** choline and phosphatidylcholine levels were detected using Choline Assay Kit and Phosphatidylcholine (PC) Colorimetric Kit after ATG 7 silencing. ***P* < 0.01, ****P* < 0.001 *versus* si-Control.

SUPPLEMENTARY FIGURE S5
Ginsenosides Rh2 and Rg3 induce upregulation of autophagic flux. **(A)** Immunoblot analysis with the LC3B antibody on cell lysates treated with TGS, ginsenosides Rh2 and Rg3 for 12 h, GAPDH serves as a loading control. **(B)** The semi-quantitative results of LC3II/GAPDH of **(A)** using the Volume Tool in Image Lab software. **P* < 0.05, ***P* < 0.01, ****P* < 0.001 *versus* control.

## Abbreviations

3-MA, 3-methyladenine; CCK-8, Cell Counting kit 8; CQ, chloroquine; ER stress, endoplasmic reticulum stress; FBS, fetal bovine serum; GAPDH, gluceraldelyde-3-phosphate dehydrogenase; MDC, monodansylcadaverine; NSCLC, non-small cell lung carcinoma; PBS, phosphate buffered saline; PBST, PBS containing 0.1% Tween-20; PMSF, phenylmethanesulfonyl fluoride; qRT-PCR, quantitative real-time polymerase chain reaction; Rapa, rapamycin; siRNA, small interfering RNA; TBST, Tris-buffered saline containing 0.1% Tween-20; TGS, total ginsenosides extract.
