# Supplementary figures and images for: Ginsenosides Rh2 and Rg3 exert their anti-cancer effects on non-small cell lung cancer by regulating cell autophagy and choline-phosphatidylcholine metabolism
Source: Front Pharmacol. 2025 Jun 12;16:1507990. doi: 10.3389/fphar.2025.1507990 (PMC12207009; doi:10.3389/fphar.2025.1507990)

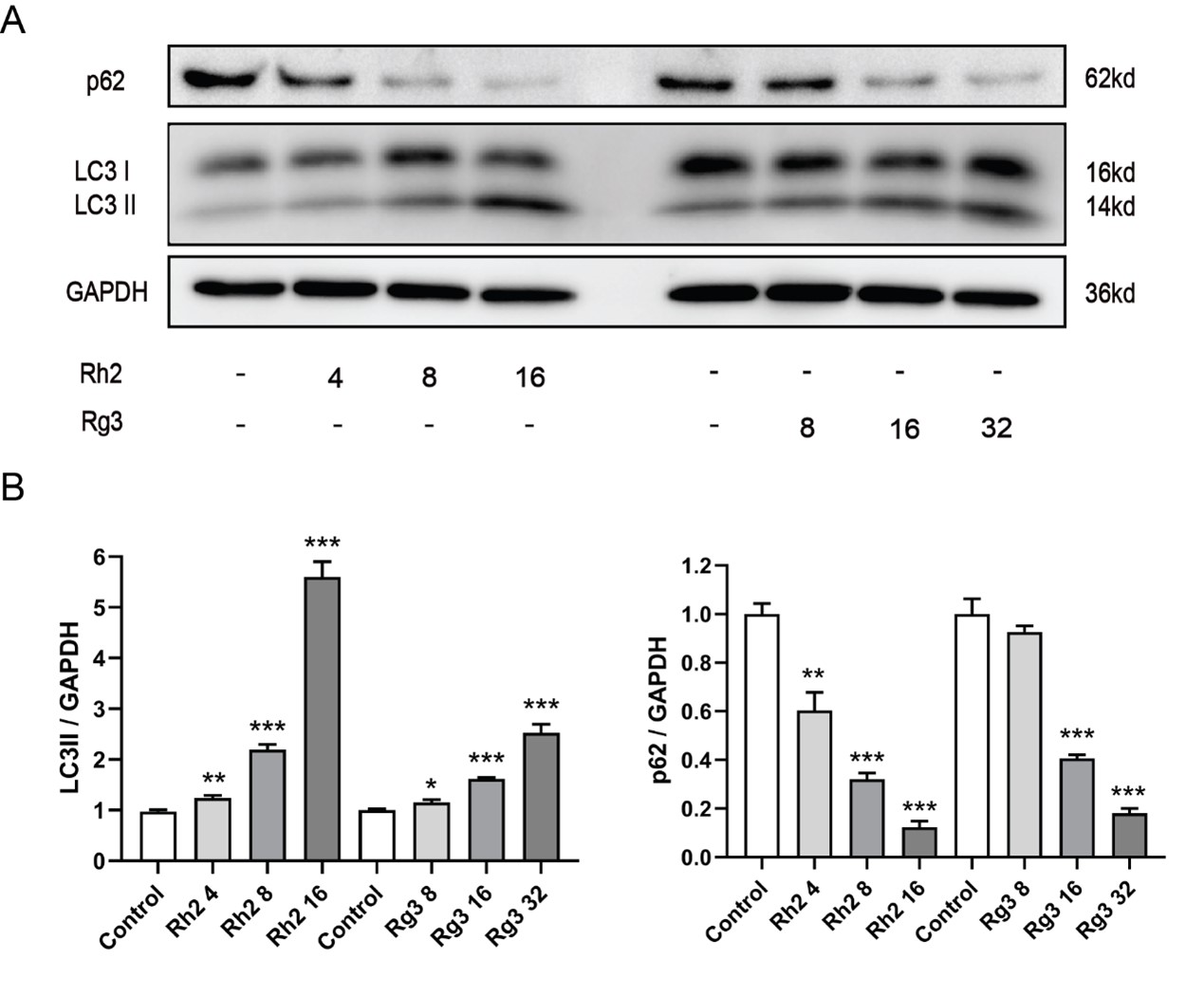

Supplement: Supplementary file 1 [file Image5.jpg]

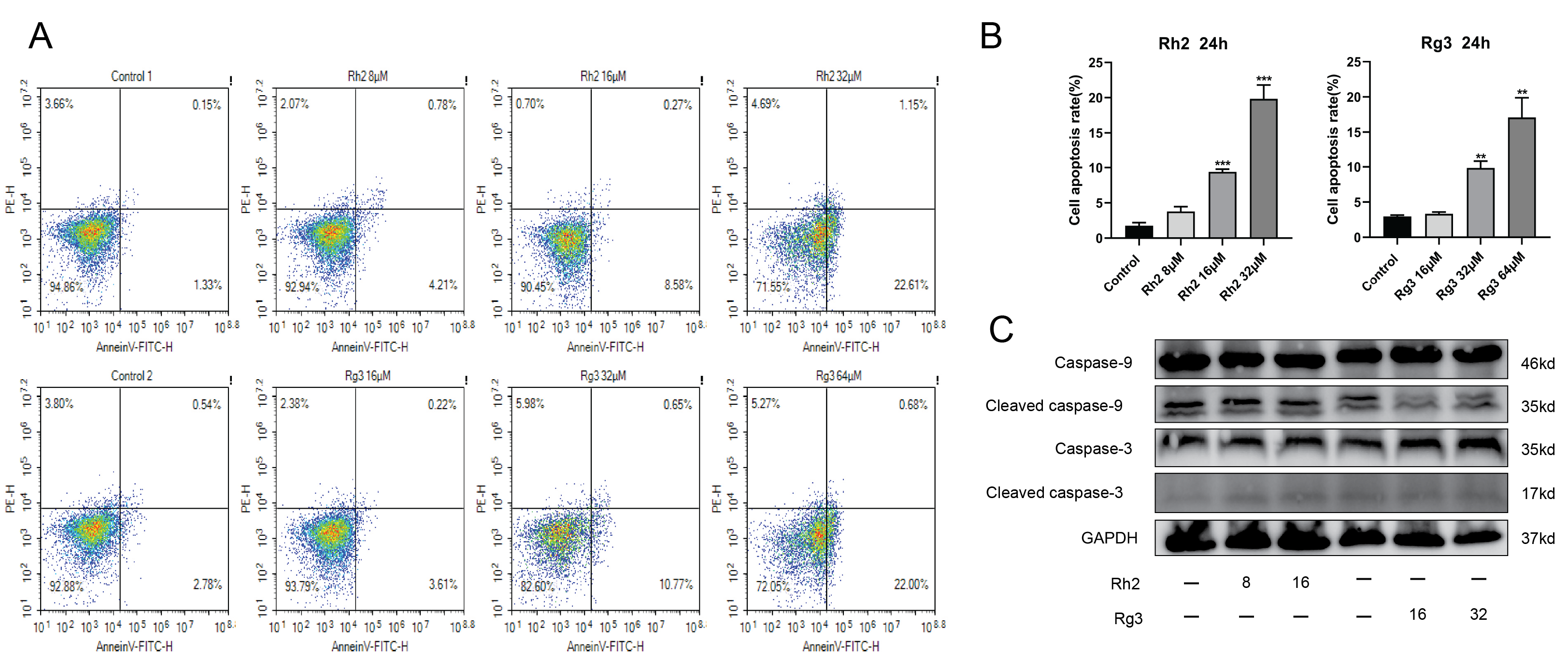

Supplement: Supplementary file 2 [file Image3.tif]

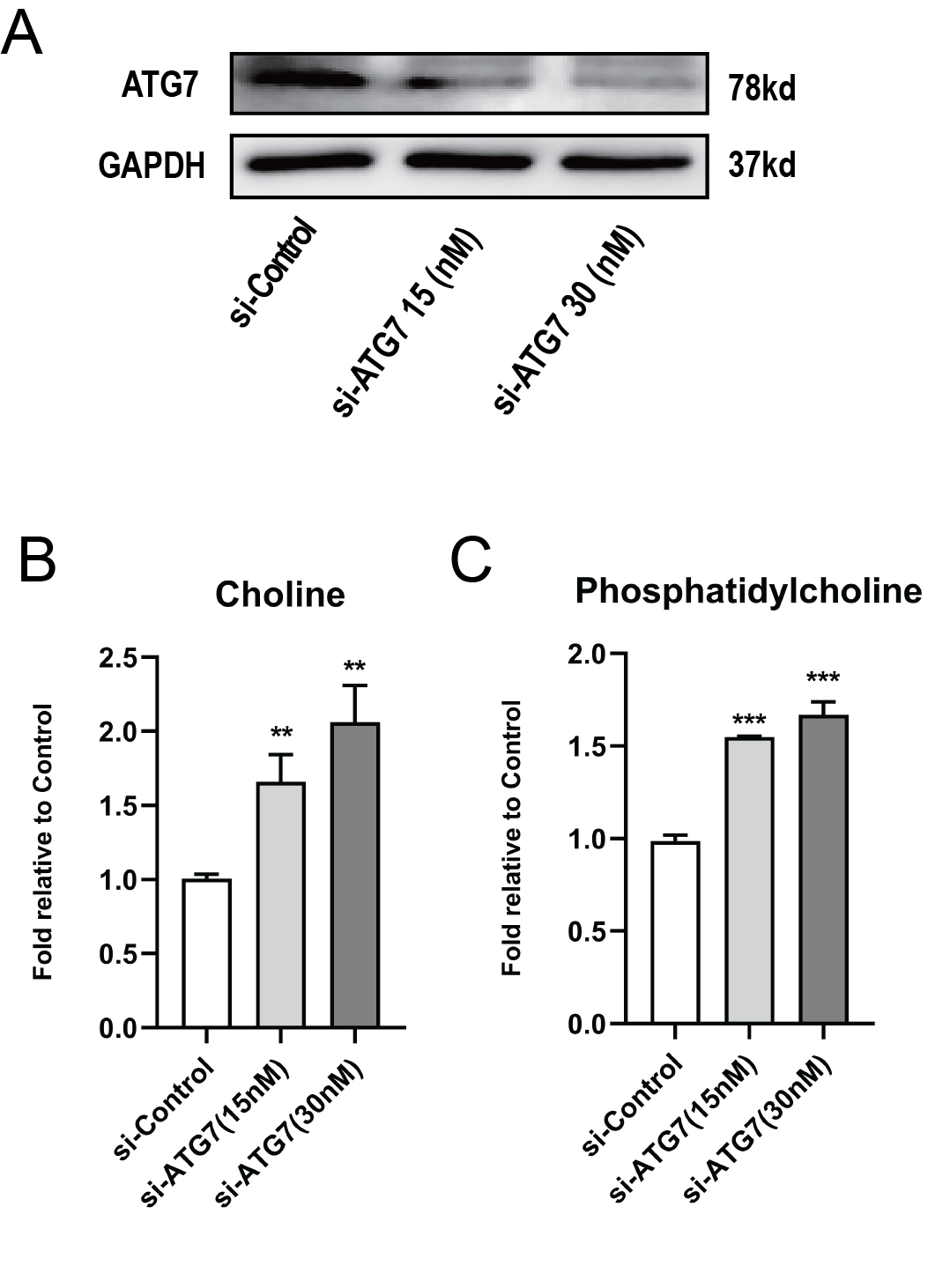

Supplement: Supplementary file 3 [file Image4.tif]

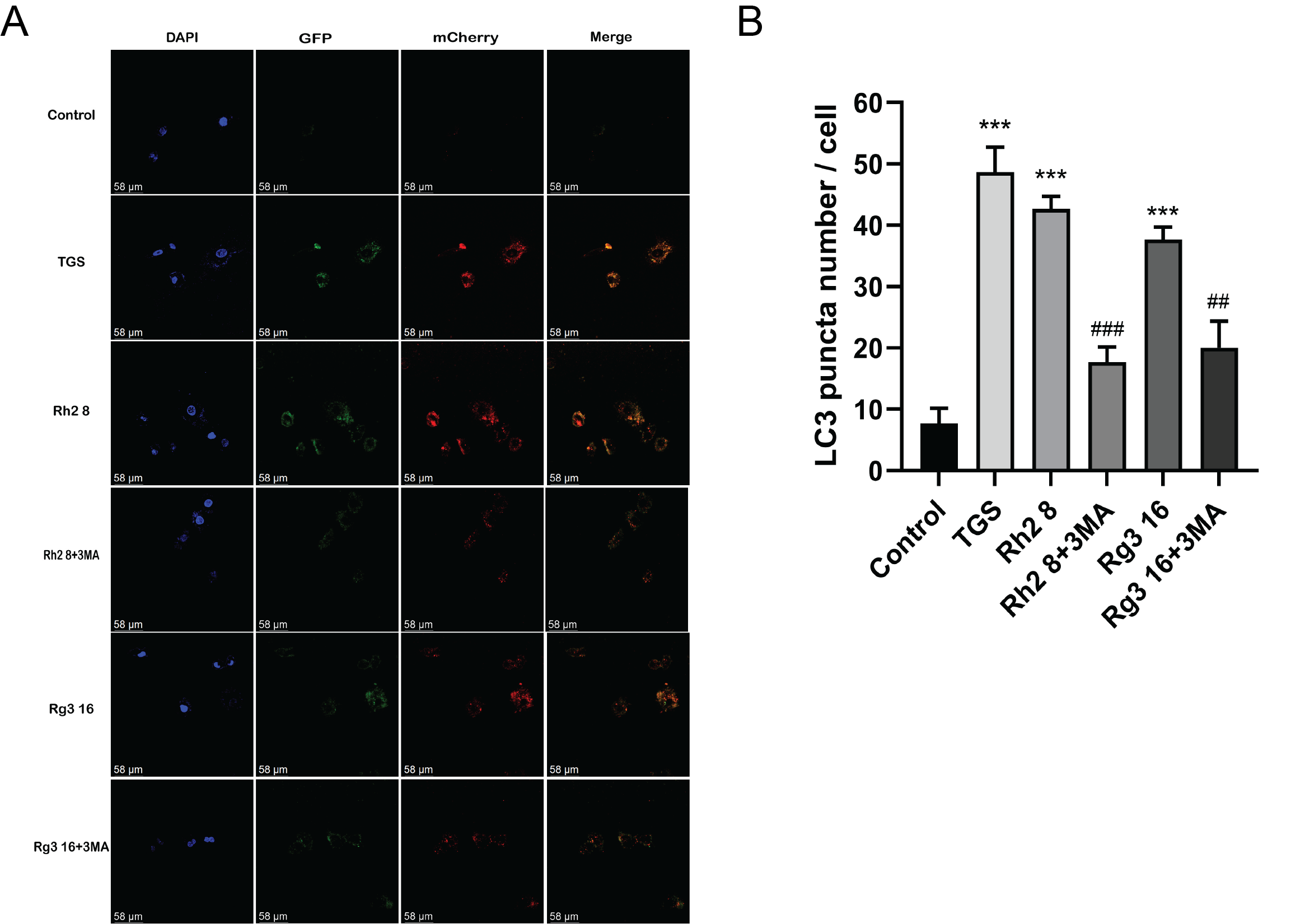

Supplement: Supplementary file 4 [file Image2.tif]

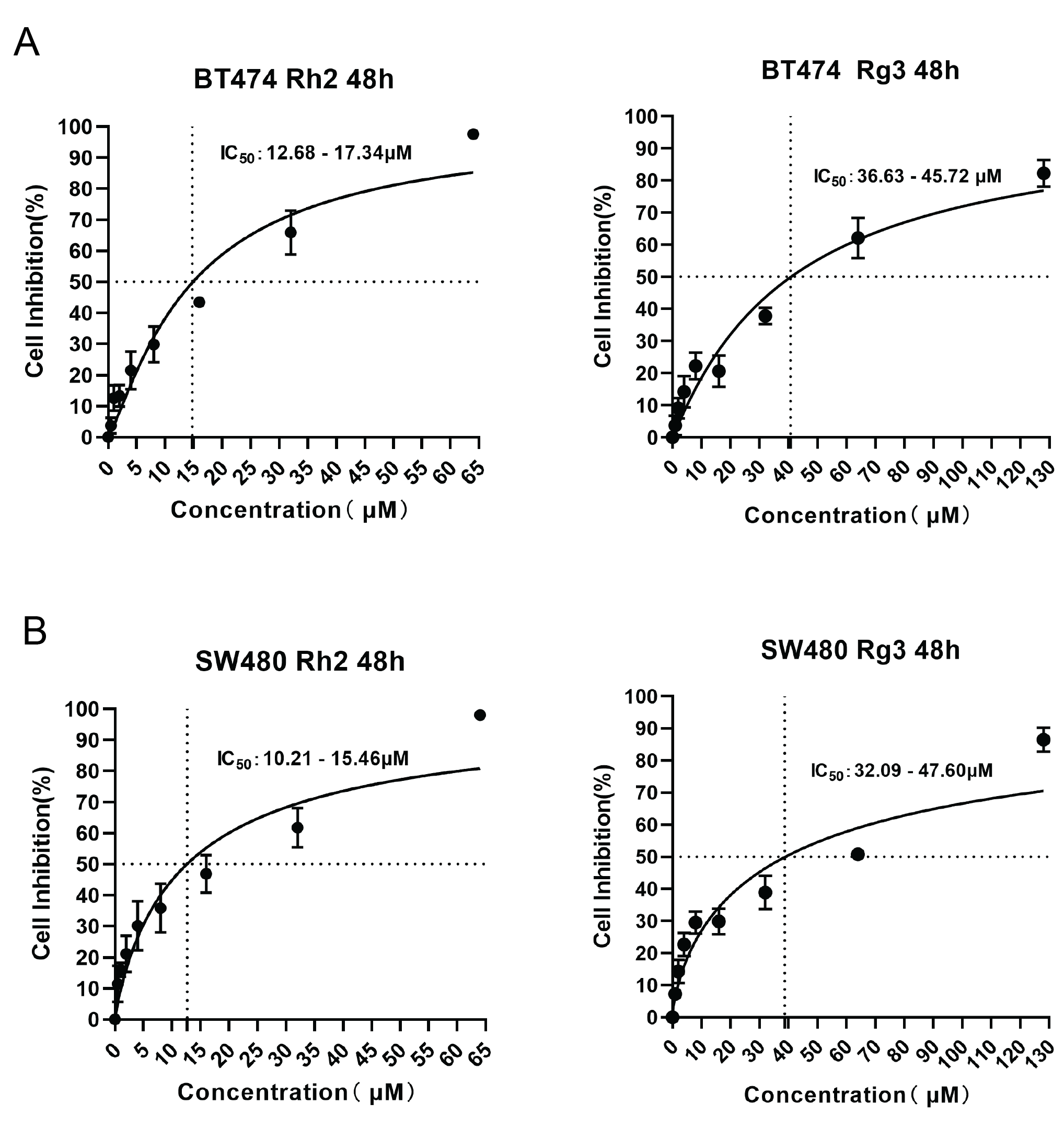

Supplement: Supplementary file 5 [file Image1.tif]
